# Removal of Silver Stains from the Hands

**Published:** 1870-01

**Authors:** 


Removal of Silver Stains from the Hands.—The *Popular Science Review* copies from the *Photographic News* the following: "Put half a pound of glauber salts, quarter of a pound of chloride of lime (the sanitary disinfectant,) and 8 ounces of water into a little wide mouthed bottle, and when required for use, pour some of the thick sediment into a saucer, and rub it well over the hands with pumice stone or a nail brush, and it will clean the fingers quite equal to cyanide, but without any danger. This will do to use over again until exhausted, and should be kept corked up. The disagreeable smell may be entirely avoided by the liberal use of lemon juice, which not only removes the smell but whitens the hands."

---

The so-called office mucilage is a watery solution of the gum-like product of the action of dilute acid upon starch, at 200 to 212 degrees F., to which some acetic acid or alcohol has been added. This gum-like product is the dextrine or British gum of commerce. There are two kinds of it, one colorable by iodine, and a dextrine not colorable by iodine—both, we suppose, equally well adapted for the purpose desired.—*Jour. of Applied Chemistry.*

